# Factors affecting executive function performance of Brazilian elderly in the Stroop test

**DOI:** 10.1590/1414-431X2022e11917

**Published:** 2022-04-27

**Authors:** P.L.G. Braga, J.S. Henrique, S.S. Almeida, R.M. Arida, S. Gomes da Silva

**Affiliations:** 1 Programa de pós-graduação em Neurologia/Neurociências, Universidade Federal de São Paulo, São Paulo, SP, Brasil; 2 Faculdade de Educação Física, Faculdade do Clube Náutico Mogiano, Mogi das Cruzes, SP, Brasil; 3 Programa de pós-graduação em Psicogerontologia, Instituto Educatie de Ensino e Pesquisa, Mogi das Cruzes, SP, Brasil; 4 Hospital Israelita Albert Einstein, São Paulo, SP, Brasil; 5 Programa de pós-graduação em Fisioterapia, Universidade Ibirapuera, São Paulo, SP, Brasil; 6 Departamento de Fisiologia, Universidade Federal de São Paulo, São Paulo, SP, Brasil; 7 Programa de pós-graduação em Engenharia Biomédica, Universidade de Mogi das Cruzes, Mogi das Cruzes, SP, Brasil; 8 Hospital do Câncer de Muriaé, Fundação Cristiano Varella, Muriaé, MG, Brasil; 9 Centro Universitário FAMINAS, Muriaé, MG, Brasil

**Keywords:** Aging, Stroop test, Physical fitness, Muscle strength

## Abstract

Aging is related to a decrease in physiological abilities, especially cognitive functions. To unravel further evidence of age-related cognitive decline, we analyzed which physical and functional variables are predictors of cognitive performance in a sample of 498 Brazilian elderly (67.26% women). To do so, we used the Stroop test as a tool to evaluate executive functions and the General functional fitness index (GFFI) to evaluate the functional fitness of the participants. A linear regression analysis revealed that female sex (β=-0.097; *t*=-2.286; P=0.023), younger age (β=0.205; *t*=4.606; P<0.0001), more years of education (β=-0.280; *t*=-6.358; P<0.0001), and higher GFFI (β=-0.101; *t*=-2.347; P<0.02) were predictors of better cognitive performance. Body mass index (kg/m^2^) and nutritional status (underweight, eutrophic, overweight, or obese) were not predictors of cognitive performance. Interestingly, among the GFFI tasks, muscle strength influenced the test execution time, both in upper and lower limbs (elbow flexion: β=-0.201; t=-4.672; P<0.0001; sit-to-stand: β=-0.125; t=-2.580; P<0.01). Our findings showed that: 1) women performed the Stroop test faster than men; 2) the older the person, the lower was the cognitive performance; 3) the higher the education, the better the test execution time; and 4) higher scores in the GFFI were associated with a better performance in the Stroop test. Therefore, gender, age, education, and functional fitness and capacity were predictors of cognitive performance in the elderly.

## Introduction

The world is facing a significant increase in the elderly population. The World Health Organization (WHO) estimates a population of 2 billion elderly people in the world by 2050, which will represent 22% of the global population, 80% of which will live in developing countries (1). In Brazil, the elderly population tripled between the 1960s and 2010, and data from WHO state that by 2025 the number of elderly will increase to 32 million, making the country the sixth largest elderly population in the world (2).

One of the major concerns of aging is the decline in functional capacity such as muscle strength, gait, balance, cognition, among others. In the elderly, loss of cognitive ability is associated with neurodegenerative diseases, which directly affect quality of life (3). Both loss of cognition and decreased quality of life are evidenced in the study by Lebrão et al. (4). Using the Mini Mental State Examination in elderlies, a prevalence of cognitive impairment of 6.9% was found, affecting 4.2% of those aged 60-74 years and 17.7% of those aged 75 years and older. In this study, it was found that the greater the cognitive loss, the greater the dependence on third parties, directly impacting quality of life. In addition, other studies show a decline in cognitive ability, as aging is associated with neuronal death, decreased plasticity, and an increase in neurodegenerative diseases (5).

Studies have highlighted the importance of preserving cognitive function during aging, especially executive function (6,7). Executive function is a set of integrated skills that enable people to direct their behaviors towards goals and carry out voluntary actions.

Several studies have shown that the older the age, the worse the score on cognitive tasks (6,8,9). Among the neuropsychological tools used to access executive functions, the Stroop test (10) is usually used. This test evaluates the ability to inhibit cognitive interference, that is, it generates a stimulus incongruity effect (11). For instance, an investigation that used the Stroop test found that there is a linear decline in executive functions with aging, regardless of processing speed, sex, and education (12). Tremblay et al. (13), when analyzing the performance of the Stroop test in the elderly, found that age was associated with worse performance in all attempts. A study conducted by Rivera et al. (6) reported that only two out of eleven countries presented differences between genders in the Stroop test. In addition to age and gender, other sociodemographic factors may be associated with performance on the Stroop test, such as schooling (14) and level of physical activity (15). Knowing that aging is associated with decreased cognitive ability and that this change can be modulated by factors such as age, gender, education, and physical fitness, the aim of the present study was to analyze physical and functional variables that can be predictive factors of cognitive performance in the elderly in Brazil. We used the General functional fitness index (GFFI) to evaluate functional fitness and the Stroop test to evaluate the executive functions of the participants.

## Material and Methods

**Figure 1:**
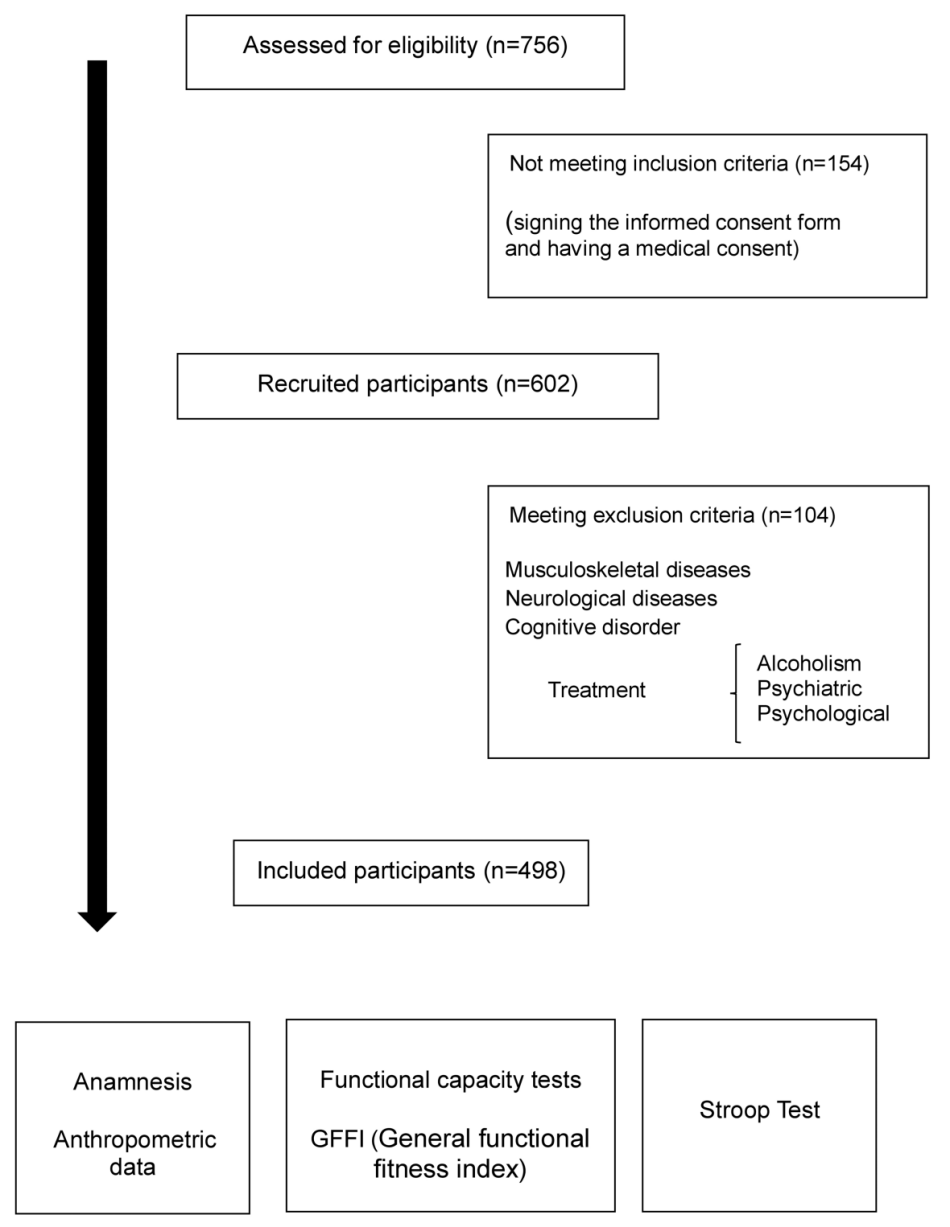
Participant selection flow chart.

We conducted a cross-sectional study with a convenience and non-probabilistic sampling that was approved by the Ethics committee of the Federal University of São Paulo (CEP/UNIFESP - CAAE 57307016.9.0000.5505). To be included in the study, participants had to sign the informed consent form (466/2012-CNS/CONEP) and have a medical consent (issued up to 1 year) to perform physical tests (requirement of elderly centers). Those who presented musculoskeletal and/or neurological diseases that made it impossible to perform the functional capacity tests or were being treated for cognitive, psychological, or psychiatric illnesses and alcoholism were excluded. A total of 756 elderly people of both sexes were recruited from elderly centers in the Alto Tiete region of São Paulo state. Of these, 154 did not meet the inclusion criterion and 104 met the exclusion criterion, leaving a final sample of 498 participants (Figure 1).

With the selected participants, the power of the study was calculated using the software G*Power version 3.1.7 (*post hoc* analysis; http://g-power.apponic.com), adopting an effect size of 25%, a 5% probability of error (P<0.05), a sample size of 498 volunteers, and 9 predictors, resulting in a power of 100%.

All evaluators and those involved in this study received prior training and performed the tests blindly.

### Education and anthropometric data

Data were collected to determine years of education. Body mass index (BMI) was determined by the following equation: BMI = Weight / (Height)^2^.

### General functional fitness index

The GFFI was initially adapted as proposed in the Senior Fit Test (16,17). The GFFI model used in this study was composed of the following tests: elbow flexion and sit-to-stand (to analyze muscular strength), time to up and go (TUG) (for analysis of sitting balance, transfer from sitting to standing, walking stability, and changing gait course), and 6-min walk test (for aerobic capacity). Tests were conducted in the following order: 1) sit-to-stand; 2) elbow flexion; 3) TUG; and 4) 6-min walk. Each functional capacity test was individually scored as very weak, weak, regular, good, and very good, stratified for every five years of life (60-64; 65-69; etc.) and gender, as proposed by Rikli and Jones (16,17). The GFFI was calculated by adding the scores of the functional capacity tests.

### Stroop test

The Stroop test was used to evaluate cognitive function. Because the studied sample contained illiterate participants, we used the validated model by Kulaif and Valle (18) with numbers and colors, adapted from the Victoria version (19). The chosen model consists of 4 cards. The first card (Card 1 - Colors) contains red, green, blue, and black rectangles that need to be quickly named. The second card (Card 2 - colors and numbers) has the numbers 4, 5, 8, and 9 in the colored rectangles as in Card 1. In this step, the participant must name the number or the color of the rectangles. The third card (Card 3 - Stroop Effect) consists of colored numbers in the same colors as card 1, and the participant must name the color of the non-black numbers, while black numbers must be named by their respective number. The participant was informed that a certain degree of interference and consequently frustration was unavoidable. A 4th card (Card 4) with numbers 4, 5, 8, and 9 colored in black was added to this model to minimize frustration. The evaluation process is based on the time the participant takes in each of the cards and the number of errors. Spontaneous self-corrected errors were considered correct answers. In order to validate the results, it was assured that the participant would recognize and name the colors without hesitation.

### Statistical analysis

The data of the Stroop test, educational level, nutritional status, functional capacities, and respective GFFI were analyzed with the general linear model (GLM) or chi-squared test. Results of GLM are reported as mean and standard deviation followed by the Fisher (F) value. The chi-squared test was conditioned to the interpretation of residuals (R=observed value minus expected value) and of the adjusted residuals (AR). The residual analysis is necessary to show which category has a significant value (P value) and the adjusted residual determines the significance level for the excess of occurrences. P values were considered significant when higher than 1.96. Added to this interpretation is the value of *X* ^2^.

Correspondence analysis was also used. This analysis consists of a graphical representation in a flat projection of multidimensional relations of *X* ^2^ distances between the categories of the studied variables. The symmetric projection was used, which allows the simultaneous examination of the relationships between rows and columns in the contingency table, that is, the relationships between all categories of both variables. Categories close to the flat projection have a stronger relationship than categories separated by greater distances. Any category, represented as a point in the projection plane, can be analyzed separately and characterized according to the proximity of the projections of all other categories, on a line that links its characteristic point to the origin of the axes of the projection plane. When categories of the same variable are found in close positions on the map of the correspondence analysis, this suggests that, regardless of their semantic content, they can be considered equal in terms of the mass distribution of the total observations made. When categories of contingent variables are projected close together, an association between the events they represent is suggested, although nothing is considered statistically significant. For statistical analysis, it is necessary to interpret adjusted residuals, as already described.

To analyze the predictive factors of the Stroop test (dependent variable - time of card 3), linear regression (stepwise model - forward) was used with the following independent variables: gender, age, nutritional status, education, and GFFI. For the analysis of which GFFI tests are predictors of the Stroop test time, linear regression (stepwise - forward model; dependent variable - time of card 3) was performed with the following independent variables: gender, elbow flexion, sit-to-stand, TUG, and 6-min walk tests. Thus, the analysis of the models adopted was done by the interpretation of the standardized and adjusted coefficient (β), followed by the analysis of the *t* and significance values from the Student's *t*-test. In addition, the interpretation of collinearity of variables is offered. If an independent variable presented more than one category, one of them was fixed to be used as reference. For example, for gender, the woman category was fixed and for the other independent variables, the last category was fixed. For all statistical tests, a level of significance lower than 5% was adopted.

## Results

Table 1 shows a descriptive analysis of the sample (gender, age, BMI, nutritional status, and education). Women made up the majority of the sample (67.26 *vs* 32.73%; *X* ^2^=59.406; P<0.0001). Non-significant differences between men and women were found for age (F=1.444; P=0.230), BMI (F=3.097; P=0.079), and nutritional status (*X* ^2^=2.094; P=0.553).

**Table 1:** Descriptive characteristics of study participants.

	General	Men	Women	AR		*X* ^2^	F	P
				Men *vs* Women				
N	498 (100%)	163 (32.73%)	335 (67.26%)			59.406		**<0.0001**
Physical characteristics								
Age (years)	71.65±6.14	72.13±6.18	71.42±6.12	-	-		1.444	0.230
BMI (kg/m^2^)	28.04±4.72	27.51±4.16	28.30±4.96	-	-		3.097	0.079
Nutritional status						2.094		0.553
Underweight	11.4	11.7	11.9	-0.1	0.1			
Eutrophic	40.3	44.8	38.8	1.0	-1.0			
Overweight	17.4	14.7	14.6	0.0	0.0			
Obese	30.7	28.8	34.6	-1.3	1.3			
Education						18.038		**<0.0001**
Never studied	21.6	14.7	25.1	-2.6	**2.6**			
1-3 years	27.7	23.9	29.6	-1.3	1.3			
4-8 years	13.7	11.7	14.6	-0.9	0.9			
9-11 years	22.7	30.7	18.8	**3.0**	-3.0			
>12 years	14.3	19.0	11.9	**2.1**	-2.1			

Data are reported as percentage (%) when categorical and by mean and standard deviation when continuous. GFFI: General functional fitness index; education >12 years: high school or more; *X* ^2^: Chi-squared; AR: adjusted and standardized residual; F: Fisher (referring to general linear model test value). Values in bold type indicate P<0.05 or positive difference in AR.

The observed frequency in men was significantly higher than expected compared to women (*X* ^2^=18.038; P<0.0001) in the following categories: 9 to 11 years of study (30.7 *vs* 18.8%) and >12 years of study (19.0 *vs* 11.9%), while in women, the observed frequency was higher than expected in the illiterate category (14.7 *vs* 25.1%; *X* ^2^=18.564 P=0.0001).

Table 2 shows GFFI and functional capacities of participants as well as an association with gender. The observed value for the categories weak, regular, and good was higher than expected (26.1, 44.6, and 24.5% respectively; *X* ^2^=319.811; P<0.0001). However, the observed frequency in men was higher than expected in the categories very weak compared to women (2.5 *vs* 0.3%; *X* ^2^=11.094; P=0.026), while women participants had a higher than expected frequency in the weak classification compared to men (19.6 *vs* 29.3%; *X* ^2^=11.094; P=0.026). When adding up the percentage of weak and regular classifications, 70.7% of the participants were in these categories.

**Table 2:** General linear model results for the General functional fitness index (GFFI) and functional capacity tests.

	General (%)	*X* ^2^	R	P	Men (%)	Women (%)	Men *vs* Women			
							AR		*X* ^2^	P
							Men *vs* Women			
GFFI		319.811		**0.0001**					**11.094**	**0.026**
Very weak	1.0		-94.6		2.5	0.3	**2.3**	-2.3		
Weak	26.1		**30.4**		19.6	29.3	-2.3	**2.3**		
Regular	44.6		**122.4**		44.8	44.5	0.1	-0.1		
Good	24.5		**22.4**		28.2	22.7	1.3	-1.3		
Very good	3.8		-80.6		4.9	3.3	0.9	-0.9		
Elbow flexion		553.888		**0.0001**					5.687	0.224
Very weak	2.8		-85.6		4.9	1.8	**2.0**	-2.0		
Weak	6.2		-68.6		7.4	5.7	0.7	-0.7		
Regular	9.8		-50.6		8.0	10.7	-1.0	1.0		
Good	20.7		**3.4**		22.1	20.0	0.5	-0.5		
Very good	60.4		**201.4**		57.7	61.8	-0.9	0.9		
Sit-to-Stand		19.851		**0.001**					**14.636**	**0.006**
Very weak	12.7		-36.6		7.4	15.2	-2.5	**2.5**		
Weak	24.1		**20.1**		19.9	26.3	-1.6	1.6		
Regular	22.5		**12.4**		21.5	23.0	-0.4	0.4		
Good	21.5		**7.4**		28.8	18.2	**2.6**	-2.6		
Very good	19.3		-91.6		23.3	17.3	1.6	1.6		
TUG		282.201		**0.0001**					9.123	0.058
Very weak	45.6		**127.4**		41.1	47.8	-1.4	1.4		
Weak	25.1		**25.4**		22.1	26.6	-1.1	1.1		
Regular	18.3		-8.6		21.5	16.7	1.3	-1.3		
Good	9.4		-52.6		14.1	7.2	**2.5**	-2.5		
Very good	1.6		-91.6		1.2	1.8	0.5	-0.5		
6-min walk		160.173		**0.0001**					5.149	0.272
Very weak	28.7		**43.4**		31.9	27.2	1.1	-1.1		
Weak	33.3		**66.4**		28.2	35.8	-1.7	1.7		
Regular	23.7		**18.4**		22.1	24.7	-0.6	0.6		
Good	11.8		-40.6		14.7	10.4	1.4	-1.4		
Very good	2.4		-87.6		3.1	2.1	0.7	-0.7		

Data are reported as percentage (%). R: Residual (observed value - expected value); *X* ^2^: Chi-squared; AR: Adjusted and standardized residual; TUG: time to up and go. Values in bold type indicate P<0.05 and positive difference in R or AR.

The observed value for the good and very good categories for strength of upper limbs assessed by the elbow flexion test was higher than expected (20.7 and 60.4%; *X* ^2^=553.888; P<0.0001), indicating the maintenance of strength. Nonetheless, a significant difference between genders (*X* ^2^=5.687; P=0.224) was found, with the observed value in men being higher than in women.

As for strength of lower limbs, measured by the sit-to-stand chair test, the observed frequencies of weak, regular, and good classifications were higher than expected (24.1, 22.5, and 21.5% respectively; *X* ^2^=19.851; P<0.0001). However, in gender comparison, the observed frequency in men was higher than expected for the good classification (28.2 *vs* 18.2%. *X* ^2^=14.636; P=0.006), while the observed frequency in women was higher in the very weak classification (7.4 *vs* 15.2%; *X* ^2^=14.636; P=0.006). These findings showed that men presented ideal values for this functional capacity while women presented values below the recommended level.

In the TUG test, observed values were higher than expected in the categories very weak and weak (45.6 *vs* 25.1%; *X* ^2^=63.380 and 282.201; P<0.0001, respectively). There was no significant difference between gender for this test (P=0.058). It is possible to conclude that this functional capacity was worse than the recommendation in our sample.

Finally, in the 6-min walk test, the observed values were higher than expected in the categories very weak, weak, and regular (28.7, 33.3, and 23.7% respectively; *X* ^2^=160.173; P<0.0001). However, no significant difference was observed between genders (*X* ^2^=5.149; P=0.272), indicating that the aerobic capacity in men and women was below the regular, presenting an unsatisfactory result.

**Figure 2:**
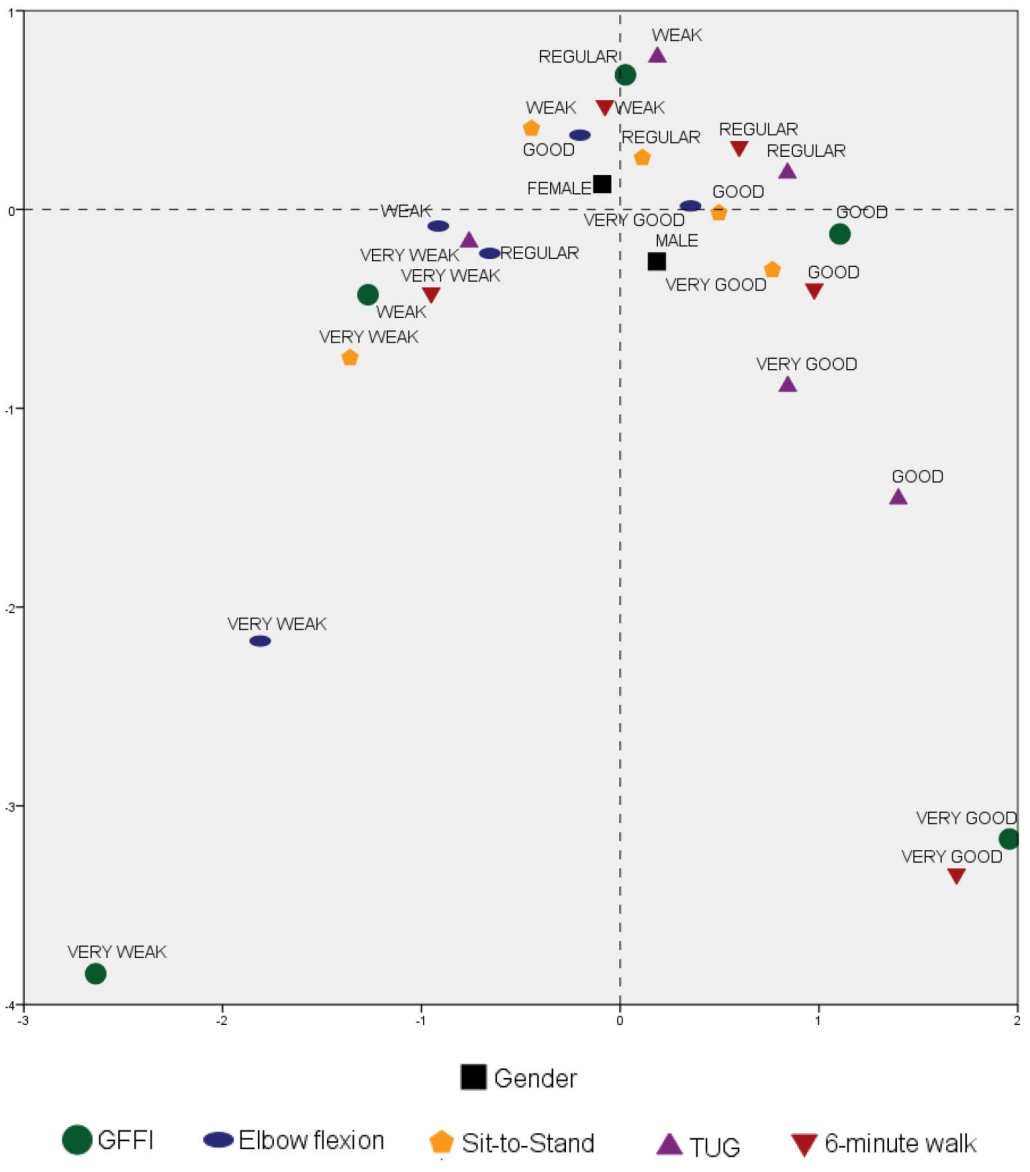
Map of the relationship between genders, GFFI classifications, and functional capacity test classifications. GFFI: General functional fitness index; TUG: time to up and go.

Figure 2 is a graphical representation of the results of Table 2. In a first analysis, it was possible to observe a representativeness of 99.6% of the variations of the quadratic-chi distances. The removal of the classifications very weak and very good for the GFFI, very weak for the elbow flexion, and very good for the 6-min walk test showed that such classifications were not common for the studied population. Another analysis was the classifications of functional capacities. As they had small distances from each other, they were considered equivalent, which supports the grouping by GFFI. Finally, Figure 2 suggests that the distribution of the variables was similar between genders, as the formation of a cloud is evident in the upper right quadrant of the map. This information adds to the study of functional capacities, as it presents a new perspective by quadratic-chi analysis.

Table 3 shows the Stroop test values, time spent at each card, errors, interference, and time spent at card 3 compared to card 1. Elderly women performed the tasks of cards 1 and 2 faster than elderly men (18.89±6.02 *vs* 20.90±10.44 s; F=7.280; P=0.007; 21.32±7.42 *vs* 23.27±12.00 s; F=4.958; P=0.026). Women had fewer errors in card 2 than men (0.31±0.9 *vs* 0.53±1.27 errors; F=4.910; P=0.027). No significant difference between genders was found for card 3 (Stroop effect), card 4, and interference neither for time nor for error counting. Yet, a significant difference was found between genders for both average time spent in card 3 compared to card 1 and error counts (F=9.265 and F=8.132; P<0.0001 for time and errors). Thus, the Stroop effect was not significantly different between genders, but gender difference was evident between different cards.

**Table 3:** Time in seconds taken to complete the card tasks, errors, and interference of the Stroop test.

Stroop test	General (498)	Men (163)	Women (335)	F_1_	P (between genders)	F_2_	P (card 3 *vs* card 1)
Card 1 (s)	19.55±7.79	20.90±10.44	18.89±6.02	7.280	**0.007**		
Errors (unity)	0.29±1.22	0.42±1.44	0.23±1.08	2.769	0.097		
Card 2 (s)	21.95±9.20	23.27±12.00	21.32±7.42	4.958	**0.026**		
Errors (unity)	0.38±1.03	0.53±1.27	0.31±0.9	4.910	**0.027**		
Card 3 (s)	43.76±19.44	45.30±21.35	43.17±18.21	1.238	0.266	9.265	**<0.0001**
Errors (unity)	2.80±3.62	2.79±3.44	2.80±3.71	0.003	0.956	8.132	**<0.0001**
Card 4 (s)	14.54±7.32	14.54±10.79	14.54±4.82	0.0	1.000		
Errors (unity)	0.07±0.48	0.09±0.54	0.06±0.44	0.265	0.607		
Interference (3-1)	23.96±15.87	23.78±16.43	24.05±15.61	0.031	0.859		

Data are reported as means and standard deviations. F_1_: univariate general linear model; F_2_: paired general linear model. Values in bold type indicate P<0.05.

Table 4 shows the predictors of performance in the Stroop test. In this model, with the analysis made by the interpretation of the standardized and adjusted coefficient (β) of the independent variables gender, age, education, and GFFI, it was observed that elderly women performed the task 0.097 s faster than elderly men (*t*=-2.286; P=0.023). Regarding age, each year of life added 0.205 s in task execution (*t*=4.606; P<0.0001). In addition, the higher the education, the less the time required to finish the test, 0.28 s faster (*t*=-6.358; P<0.0001). Finally, the higher the GFFI, the less the time required to complete the task, 0.10 s faster (*t*=-2.347; P=0.019). Nutritional status was excluded from the model for not being a predictor for the Stroop test performance in the study population. In brief, these findings show that gender (women), age (younger), schooling (higher), and GFFI (higher) were predictors for better performance in the Stroop test.

**Table 4:** Predictive factors of Stroop test performance.

	Adjusted model			Tolerance
	β	*t*	P	
Gender	-0.097	-2.286	**0.023**	0.96
Age	0.205	4.606	**<0.0001**	0.86
Education	-0.280	-6.358	**<0.0001**	0.88
General functional fitness index	-0.101	-2.347	**0.019**	0.92

Adjusted linear regression model (β): stepwise method. Dependent variable: time taken for card 3. Independent variables (fixed): gender (woman as reference), nutritional status (obese as reference), schooling (>12 years of education as reference); General functional fitness index (very good as reference). Nutritional status was not a significant predictor. Values in bold type indicate P<0.05.

Since the GFFI is composed of four distinct tests, and these combined with gender contribute to the composition of the index, we enlightened the concept of functional capacities and gender being predictors of performance in the Stroop test (Table 5). Elbow flexion and the sit-to-stand tests were predictors of performance, in which the better the performance on these tests, the lower the time needed to perform the Stroop test: 0.201 s faster (*t*=-4.672; P<0.0001) for elbow flexion and 0.125 s faster (*t*=-2.580; P=0.01) for the sit-to-stand test. Gender and functional capacities tests (TUG and 6-min walk) were excluded from the model for not being predictors of Stroop test performance. Finally, our findings suggested that muscle strength (both in upper and lower limbs) was a predictor of Stroop test performance.

**Table 5:** Predictive factors of Stroop test performance in relation to the components of General functional fitness index.

	β	t	P	Tolerance
Elbow flexion	-0.201	-4.672	**<0.0001**	0.94
Sit-to-Stand	-0.125	-2.580	**0.01**	0.94

Adjusted linear regression model (β): stepwise method. Dependent variable: time taken for card 3. Independent variables (fixed): gender (woman as reference), elbow flexion, sit-to-stand, time to up and go, and 6-min walk test (very good as reference). Gender, time to up and go, and 6-min walk test were not significant predictors. Values in bold type indicate P<0.05.

## Discussion

Aging is associated with a decrease in physiological capacities, especially functional and cognitive abilities. In the present study, we aimed to analyze predictive factors for cognitive performance in a Brazilian elderly sample using the Stroop test as a tool to evaluate selective attention and executive functions. Our results indicated that gender (women), age (younger), education (higher), and GFFI (higher) were predictive factors for the Stroop test performance. Moreover, among the tests of the GFFI, only muscle strength was a potential predictor for Stroop test performance.

In the social context, the current findings suggested that age and educational level were crucial factors when planning programs for cognitive rehabilitation. Furthermore, the more the years of education, the greater the gain in cognitive function. Our results were consistent with other studies (20). In the study by Tian et al. (21), female gender showed significantly better executive function, attention, and memory compared to male gender. However, there is still no consensus in the literature, as in the study by Rivera et al. (6), where only two out of eleven countries showed differences between genders in the Stroop test.

Another predictor of cognitive function was functional fitness. Other studies show similar results, where physically active elderly women showed better executive function compared to men (22,23). In addition, factors such as lifestyle and physical and functional performance, especially at the end of adult life and the beginning of old age, are directly related to cognitive performance, especially executive functions (24). Thus, the data confirmed that the higher the functional fitness, the better the preservation of cognitive functions.

Among the functional capacities, upper limb strength was preserved in both genders in our sample. Data regarding this subject are controversial. For instance, our data differ from the study by dos Santos et al. (25) in which the healthy elderly group was classified in the very weak category. However, in a study by Souza et al. (26), in which elderly subjects underwent an eight-week functional training program, the volunteers made an average of 33.90±4.09 movements, which is considered very good for the age.

As for lower limb strength, elderly men had better than expected values, while more women were included in the very weak category. The association between lower limb strength and the probability of hospitalization has been reported, showing that participants with weaker legs have a higher prevalence of hospital admission (27).

Participants of both sexes were classified as very weak and weak in the TUG test. The TUG test has been widely used in clinical practice and in research for the evaluation of sarcopenia, survival, functional mobility, and risk of falls in the elderly community (28). Elderlies with values below the recommended have greater levels of fragility and morbidity (29).

The results of the 6-min walk test showed that the aerobic capacity of participants of both sexes was unsatisfactory. Similar to TUG, the 6-min walk test has been widely used as a scientific instrument because of its high correlation with morbidity/mortality (30). Cahalin et al. (31) found that the probability of death/hospitalization in individuals who walked less than 300 m in 6 min was high. In addition, in a study with participants aged 68.9±5.4 years, women walked 521.7±71.5 m while men walked 584.2±82.6 m, which was significantly more. In addition, both these values would be considered weak in our protocol. Our data corroborated those findings, since women presented worse performance compared to men.

Since GFFI was a predictor of executive functions, we sought to verify which of the GFFI tests was contributing to the result. Muscle strength was the only factor associated with performance in the Stroop test. Unlike our results, the literature shows that both acute aerobic and strength exercises improve executive functions (30,32). However, there is an increasing number of studies showing the relationship between higher muscle strength and improvement of cognitive function. In older women, quadriceps strength is associated with executive functions (i.e., attention/working memory), independent of aerobic fitness (33). Elderly women who practiced resistance training and had increased muscle strength had a significantly better performance on the Stroop test (34). Elderly women with a greater rate of torque had faster simple tapping speed and better language performance. Moreover, a greater rate of velocity development in the upper extremity was associated with better executive functions, attention, and memory (12). In addition, higher levels of physical functioning (especially muscle strength) is associated with improved cognition in healthy older adults (35).

Several studies have found that sarcopenia is associated with decreased cognitive ability (4,36). In addition, elderly people who have lower muscle mass index (muscle mass-to-body surface ratio) have lower cognitive function (37). Furthermore, our results were similar to those of Smolarek et al. (38), who found that significant gains in muscle strength are associated with cognitive improvements in the elderly.

Finally, the majority of studies address levels of physical fitness through questionnaires (a subjective assessment) or isolated functional capacity tests, rather than through an index. In this study, we used an index and the classification proposed by Rikli and Jones (16,17), as mentioned previously. In this way, the natural aging process and the gradual decline in functional capacity are taken into account when classifying individuals (e.g., the result of 17±6.3 movements in the elbow flexion test for 64-year-old men is classified as regular, while it is classified as good for 75-year-olds).

The limitations of our study should be mentioned to guide future investigations. First, there were no tools to equalize the initial cognitive status of participants or even factors that could interfere with their cognitive function. Second, there is a lack of instruments available for cognitive assessment, and it would be interesting to associate multiple tests to assess executive cognitive function or tasks for other cognitive domains (e.g., processing speed). However, due to the number of participants and research volunteers that worked in this study, we were unable to perform further cognitive analyses. The average time spent applying the tests was 60 min per person, with 30% of this time used for the Stroop test.

Nevertheless, our results showed that GFFI was a predictor of executive capacity as measured by the Stroop test. We also observed that muscle strength played an important role in executive function. This information adds to the existing literature and reinforces the attention that should be paid to the reduction of muscle mass and especially muscle strength in the elderly, as they are much more prone to sarcopenia. In this sense, the present study indicated an interesting association between muscle strength and cognitive capacity. These findings are also relevant for healthcare professionals, since they indicate the importance of maintaining functional capacities (especially muscle strength) for the maintenance of cognitive function in the elderly. Improving executive functions can be a valuable asset in the quality of life of older adults. Once predictors of cognitive performance are known, the scientific and medical communities will have more accurate subsidies for the treatment/prevention of cognitive decline.
